# Unbalanced 2D Chiral Crystallization of Pentahelicene Propellers and Their Planarization into Nanographenes

**DOI:** 10.1002/chem.202101223

**Published:** 2021-06-08

**Authors:** Jan Voigt, Myriam Roy, Miloš Baljozović, Christian Wäckerlin, Yoann Coquerel, Marc Gingras, Karl‐Heinz Ernst

**Affiliations:** ^1^ Surface Science and Coating Technologies Empa Swiss Federal Laboratories for Materials Science and Technology Überlandstrasse 129 8600 Dübendorf Switzerland; ^2^ Aix Marseille Univ. CNRS CINAM Marseille France; ^3^ Aix Marseille Univ. CNRS Centrale Marseille iSm2 Marseille France; ^4^ Nanosurf Laboratory Institute of Physics The Czech Academy of Sciences Cukrovarnická 10 16200 Prague Czech Republic; ^5^ Department of Chemistry University of Zurich 8057 Zurich Switzerland

**Keywords:** graphene, helicenes, on-surface chemistry, polycyclic aromatic hydrocarbons, scanning tunneling microscopy

## Abstract

The chiral self‐assembly of trispentahelicene propellers on a gold surface has been investigated in ultrahigh vacuum by means of scanning tunneling microscopy and time‐of‐flight secondary ion mass spectrometry. The trispentahelicene propellers aggregate into mirror domains with an enantiomeric ratio of 2 : 1. Thermally induced cyclodehydrogenation leads to planarization into nanographenes, which self‐assemble into closed‐packed layers with two different azimuths. Further treatment induces in part dimerization and trimerization by intermolecular cyclodehydrogenation.

## Graphical abstract

**From helical to planar**: Molecular propellers built up by three pentahelicene subunits self‐assemble on a gold surface into enantiomerically unbalanced two‐dimensional crystal domains. Upon thermal treatment of the close‐packed monolayer, intramolecular cyclodehydrogenation into planar nanographenes occurs. Further thermal treatment leads in part to fusion into larger graphene flakes of up to four nanographene units by intermolecular cyclodehydrogenation.

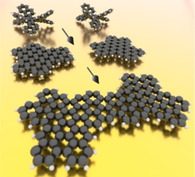

Racemic mixtures of chiral molecules crystallize either into racemic crystals with both enantiomers in the crystal unit cell or into a conglomerate of homochiral crystals, that is, a single crystal that contains only molecules of identical handedness. Other possibilities are random distribution of enantiomers, so‐called solid solution (or pseudoracemate),^[1]^ or chiral twinning into enantiopure laminates.^[2]^ Although observed for more than 170 years, the outcome of chiral crystallization is yet unpredictable. There have been early attempts of explanation, which were later shown to be biased by resolvable compounds.^[3]^

In order to better understand the complex phenomenon of chiral crystallization, well‐defined two‐dimensional (2D) model studies of chiral molecule aggregation on surfaces have been increasingly pursued in the last two decades.^[4]^ In particular the application of scanning tunneling microscopy (STM) with its submolecular resolution allowed valuable insights.^[5]^ One interesting class of molecules studied have been helical aromatic hydrocarbons, so‐called helicenes.^[6]^ Their surface self‐assembly has been especially motivated by physical phenomena, such as electron spin selectivity, molecular electromechanics and chiroptical responses.^[7]^

Concerning the outcome of chiral crystallization, heptahelicene ([7]H) for example formed zigzag rows with alternating *(M)*/*(P)* enantiomers on the three (111) surfaces of Cu, Ag and Au,^[8]^ but a 2D conglomerate was formed on Cu(100).^[9]^ A special, coverage‐dependent transition from homochiral clusters into racemic phases has been observed for [7]H and trioxa[11]helicene on Ag(100),^[10]^ while a conversion from a solid solution into a racemate was reported for 5‐amino[6]helicene by alloying the Cu(100) surface with Sn.^[11]^ Interestingly, functionalization of [7]H with cyano groups causes conglomerate formation on Cu(111),^[12]^ while bromo‐, benzo‐ and S‐acetyl‐functionalized [7]H showed again the racemic zigzag motif on Ag and Au(111) surfaces.^[13]^

Here it is shown that the 2D crystallization of the *D*_3_‐symmetric tris(pentahelicene)benzene (T[5]H) leads to mirror domains on the Au(111) surface that have 33 % enantiomeric excess. That is, the enantiomeric *M*/*P*‐ratio is 2 : 1 in one mirror domain and 1 : 2 in the other. **T[5]H** represents a hexapole helicene, containing six embedded pentahelicene subunits (Scheme 1).^[14]^ In contrast to single pentahelicene (**[5]H**), the *(outer‐M)‐* and *(outer‐P)‐*enantiomers have a much higher barrier for enantiomerization (150 kJ/mol). As previously shown for single molecules, thermal treatment leads to planarization by partial dehydrogenation into a nanographene (**NG**, Scheme 1).^[15]^ The intramolecular – as well as partial intermolecular – hydrogen abstraction is confirmed by means of time‐of‐flight secondary ion mass spectrometry (ToF‐SIMS).

**Scheme 1:**
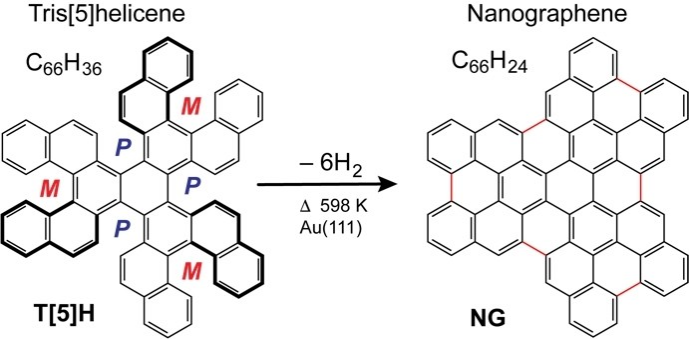
Structure models of tris[5]helicene (T[5]H) and its dehydrogenation product trisperylenocoronene (nanographene, NG). The *(outer‐M)*‐enantiomer of **T5H** is displayed. Note that the three inner pentahelicene units have *(P)*‐helicity. From enantiomerically unbalanced domains in the monolayer dehydrogenation into NG layers with two different azimuthal orientations occurs.

**T[5]H** has been synthesized as described previously.^[14b]^ The Au(111) sample was cleaned by Ar^+^ ion sputtering and annealing. Racemic **T[5]H** was thermally sublimed (350 °C, p=5×10^−10^ mbar) onto the Au sample kept at room temperature (RT). STM was also measured at room temperature. For more experimental details see Supporting Information.

Figure 1 shows STM images of single domains of self‐assembled **T[5]H**, in part superimposed with color‐coded molecular models (yellow stands for *(outer‐P)*‐**T[5]H** and red for *(outer*‐*M)*‐**T[5]H**). The surface still shows its herringbone reconstruction underneath the molecular layer. A single molecule exhibits a pinwheel STM contrast from which the absolute handedness can be deduced (Figure 1a). In general, a central **T[5]H** molecule is surrounded by six **T[5]H** molecules of opposite handedness, leading to a rhomboidal unit cell (Figure 1b). Quite unique, the unit cell shows an enantiomeric ratio of 2 : 1, which translates into a enantiomeric excess (ee) of 33 %. Including some degree of imperfection, i. e. not in all cases a next neighbor molecule has opposite handedness, a count at larger scale results in ee≈30 % (Figure 1c, d). Consequently, there are domains that have either enantiomer as majority. These have in addition an opposite oblique tilt with respect to the high symmetry directions of the substrate. In Figure 1, the majority is composed of *(outer‐P)*‐**T[5]H**. It is pointed out that in 2D chiral systems regular domains with enantiomeric excess are extremely rare.^[4a]^ Here, it is apparently a way to achieve the densest packing in the molecular layer.

**Figure 1:**
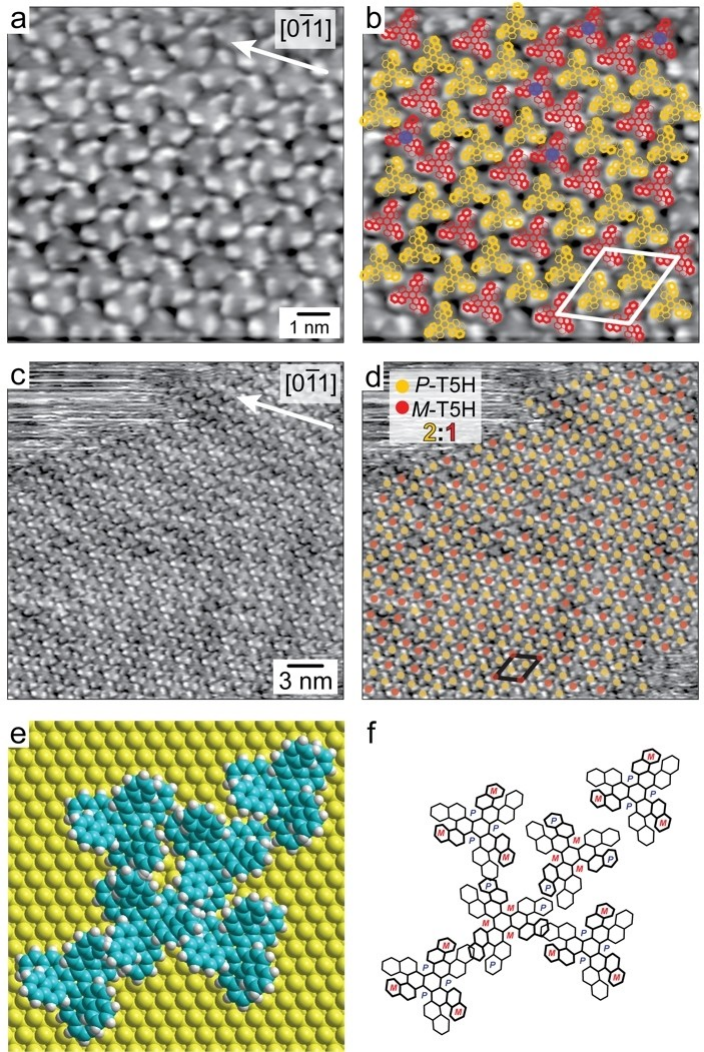
STM images (see Supporting Information for parameters) of self‐assembled T[5]H molecules at monolayer coverage on Au(111). (a) High resolution STM image. A white arrow denotes a high symmetry direction of the (111) surface. A single molecule appears as windmill, allowing assignment of absolute configuration. (b) Corresponding STM image with superposition of molecular models (yellow: *(outer‐P)*‐T[5]H, red: *(outer‐M)*‐T5H). The unit cell is indicated by a white rhomb. It contains 2 *(outer‐P)*‐T[5]H and 1 *(outer‐M)*‐T5H. The *(outer‐M)*‐T5H at the corners of the unit cell are separated by 2.36 nm. The blue dots mark deviations from the opposite‐handedness‐scheme of adjacent molecules. (c, d) Overview STM images including in part annotation with colored dots with same code as in (b). The Au(111) herringbone reconstruction underneath the molecular layer contributes to the STM contrast. (e) Full‐space model of molecular arrangement in the unit cell. (f) Adaptation of model shown in (e) with carbon frame only. The molecular unit cell is commensurate to the Au(111) surface (see Supporting Information Figure S1 for more details on the unit cell).

Recently, a stereoselective influence of coverage in a dehydrofluorization reaction of tetra(pentafluorophenyl)‐porphyrin has been reported.^[16]^ As planarization by partial dehydrogenation for isolated **T[5]H** molecules on Au(111) has been reported previously,^[15]^ it is interesting to evaluate the dehydrogenation reaction also in saturated layers, i. e., under close packing conditions. In particular ToF‐SIMS is ideally suited to identify dehydrogenation products and chemical transformation of larger molecules.^[17]^ Figure 2 shows positive ion ToF‐SIM spectra recorded after stepwise annealing of a monolayer **T[5]H** on Au(111). The **T[5]H** and **NG** mass region and the mass regions for **NG** dimers and **NG** trimers, linked by intermolecular dehydrogenation, are shown in Figure 2. The expected isotopic distributions of peaks are plotted above the spectra as black rods.^[18]^ Dotted lines accentuate the maximum peak position of each isotopic distribution. After deposition with the sample kept at room temperature, only **T[5]H** monomers (C_66_H_36_) are observed (Figure 2a, blue trace). Note that the SIMS‐induced dehydrogenation tail of **T[5]H** extends exactly to the mass of the dehydrogenation product **NG**. This is a very clear example of structurally aware SIMS‐induced dehydrogenation. Annealing the sample to 620 K results in mass peaks congruent with C_66_H_24_, i. e. **NG** molecules. Further annealing results in a progressively decreasing **NG** signal.

**Figure 2:**
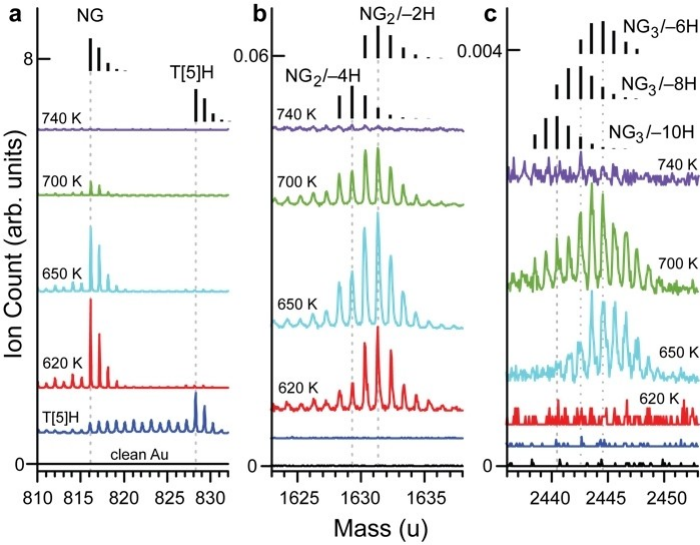
ToF‐SIM spectra of **T[5]H** on Au(111) and after stepwise annealing to the indicated temperatures. The expected isotopic mass distributions of the relevant species are shown as black bars. (a) Mass region of **T[5]H** molecules and its dehydrogenation product **NG**. The formation of **NG** after annealing to 620 K is evident from appearance of the series of peaks at corresponding masses. For all species, the series of peaks at lower masses with respect to the main peak of the species is attributed to SIMS‐induced dehydrogenation. (b) Mass region of **NG** dimers. The spectra are composed of a mixture of **NG_2_/**−**2H** (C_132_H_46_, dimerization *via* one new C−C bond) and **NG_2_/**−**4H** (C_132_H_46_, dimerization *via* two new C−C bonds). (c) Mass region of adducts of 3 **NG** molecules. The spectra are composed of a mixture of **NG_3_/**−**6H** (C_198_H_66_), **NG_3_/**−**8H** (C_198_H_64_) and – after annealing to 700 K – **NG_3_/**−**10H** (C_198_H_62_).

Dimers of **NG** (Figure 2b) are also observed after annealing to 620 K. The 620 K spectrum is mainly characterized by C_132_H_46_, i. e. dimerization of 2 **NG** molecules occurred after abstraction of two 2 H atoms (designated as **NG_2_/**−**2H**). Annealing to 650 K and 700 K results in increased intensities of masses lowered by 2 and 4 u, indicating that the sample is composed of a mixture of C_132_H_44_ (**NG_2_/**−**4H**) and C_132_H_46_ **NG_2_/**−**2H**).

**NG** trimers are observed after annealing to 650 K and 700 K (Figure 3c). The spectrum recorded after annealing to 650 K is best described by a convolution of C_198_H_66_ (**NG_3_/**−**6H**, mass_max_≈2444.5 u) and C_198_H_64_ (**NG_3_**−**8H**, mass_max_≈2442.5 u). Additional peaks at lower masses due to C_198_H_62_ (**NG_3_/**−**10H**, mass_max_≈2440.5 u) arise after annealing to 700 K. As these peaks appear exclusively after annealing, they are not caused by SIMS‐ induced dehydrogenation.

**Figure 3:**
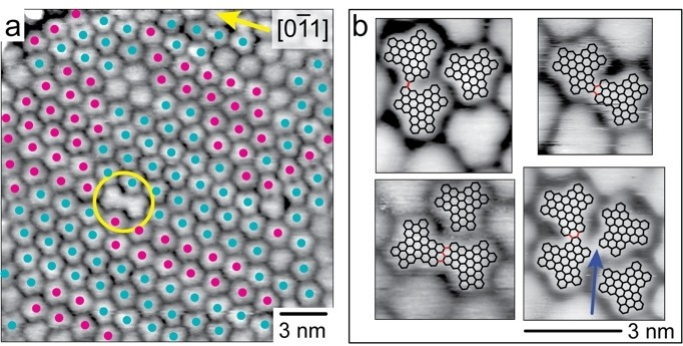
STM images (see Supporting Information for parameters) recorded after annealing a monolayer of **T[5]H** to 700 K. Planar NG molecules and dimer species formed by intermolecular C−C coupling are observed. (a) The vector of the molecular lattice is oriented parallel to the crystallographic directions of the Au(111) surface (yellow arrow). Two different orientations are identified (annotated with cyan and magenta filled circles) in which a long axis of the molecules points into opposite directions. The yellow circle marks a C−C‐coupled dimer. (b) STM images showing different dimer configurations. The blue arrow marks a very small intermolecular distance which is still too far for C−C coupling.

The adducts **NG_2_/**−**2nH** and **NG_3_/**−**2nH** observed in ToF‐SIMS correspond to **NG** dimers and trimers formed by *n*‐fold intermolecular C−C coupling. That is, dimers are linked by a one or two new C−C bonds and trimers can be linked by 3, 4 or 5 new C−C bonds. Very weak SIMS signals, corresponding to **NG** tetramers are shown in (Supporting Information Figure S2).

STM data recorded after annealing a saturated layer of **T[5]H** to 700 K are shown in Figure 3. The molecules appear planar without intramolecular contrast. In the self‐assembled layer of **NG** the molecules are aligned parallel to the high symmetry directions of the underlying surface. In addition, a molecular long axis can point into opposite directions (annotated with cyan and magenta filled circles (Figure 3a). Small domains of molecules with identical azimuthal orientation are observed, but apparently the different orientations allow equally dense packing. Superposition with molecular models exactly match the structure of **NG** (Figure 3b).

Occasionally, fused products, formed by dehydrogenation and intermolecular cyclodehydrogenation are observed. Figure 3b classifies different adducts. In the superposition, newly formed intermolecular C−C bonds are indicated in red. For each STM image, different bonding motifs were considered and the geometrically best fit in superposition was chosen. The adducts are assigned to **NG_2_/**−**2H** (Figure 3b, top left), **NG_2_/**−**4H** (Figure 3b, top right and bottom right) and NG_2_/−6H (Figure 3b, bottom left). As their abundancy is extremely low, the ToF‐SIMS‐identified trimers and tetramers, were not found in STM. The observed dimers, however, qualitatively reproduce the **NG_2_/**−**2H** and **NG_2_/**−**4H** identified by ToF‐SIMS. Interestingly, the mass of the STM‐observed **NG_2_/**−**6H** species has low SIMS intensity and remains a rare case. Note that Figure 3b (blue arrow, bottom‐right) shows a case where two molecules are too close to be uncoupled **NG** molecules but too far away for C−C coupling. This case may represent the situation in which two dehydrogenated radicals form organometallic bonds to a single Au atom.^[19]^

In conclusion, **T[5]H** 2D aggregation is one of the rare examples in which an ordered lattice shows an unbalanced enantiomeric ratio of 2 : 1. Thermal annealing of a complete monolayer leads to planarization by intramolecular dehydrogenation into nanographene plus partially intermolecular dehydrogenation and C−C coupling. As graphene formation is usually an atom‐by‐atom growth process, the fusion of nanographenes could be an alternative approach to graphene layers with tailored defect structures.

## Conflict of interest

The authors declare no conflict of interest.

## Supporting information

As a service to our authors and readers, this journal provides supporting information supplied by the authors. Such materials are peer reviewed and may be re‐organized for online delivery, but are not copy‐edited or typeset. Technical support issues arising from supporting information (other than missing files) should be addressed to the authors.

Supplementary
