# Supplementary material for: Unbalanced 2D Chiral Crystallization of Pentahelicene Propellers and Their Planarization into Nanographenes
Source: Chemistry. 2021 Jun 8;27(40):10251–4. doi: 10.1002/chem.202101223 (PMC8362048; doi:10.1002/chem.202101223)
Supplement: Supplementary file 1 — Supplementary [file CHEM-27-10251-s001.pdf]

Chemistry–A European Journal

Supporting Information

Unbalanced 2D Chiral Crystallization of Pentahelicene Propellers and Their Planarization into Nanographenes

Jan Voigt, Myriam Roy, Miloš Baljozović, Christian Wäckerlin, Yoann Coquerel, Marc Gingras, and Karl-Heinz Ernst*

Table of Contents

Experimental Procedures..2
Results and Discussions...2

Experimental Procedures

All experiments were performed on Au(111) single crystals under ultrahigh vacuum conditions. The surface was cleaned by cycles of Ar^+ sputtering and annealing. Samples were prepared by thermal sublimation of racemic tris(pentahelicene)benzene (T[5]H) on the clean Au(111) surface from an effusion cell evaporator. Typical evaporation parameters for a monolayer coverage were 350°C for one hour. Throughout preparation and analysis, the sample was kept at room temperature (RT). Scanning tunneling microscopy (STM) experiments were conducted with a Specs Aarhus 150 in constant current mode using a tungsten tip. All STM images are calibrated using atomic resolution on Cu(100). Time-of-flight secondary ion mass spectrometry (ToF-SIMS) was performed with an IONTOF ToF-SIMS 5 instrument on *in-situ* prepared samples using a 25 keV beam of Bi_3^+ primary ions. The beam was randomly rasterized over an area of 0.25 mm^2 and an extraction voltage of 3 kV was used. The mass calibration was performed using the Au_n^+ signals of the substrate. The intensity of the spectra was normalized to the Au_3^+ peaks. The pressure during preparation and measurements was below 10^{-8} mbar.

Results and Discussion

Table S1 Imaging parameters. The images were obtained at 300 K.

Figure	Bias Voltage (V)	Tunneling current setpoint (nA)
1a & 1b	1.10	0.12
1c & 1d	1.10	0.12
3a	0.80	0.21
3b top left	0.80	0.21
3b top right	1.00	0.18
3b bottom left	1.10	0.22
3b bottom right	0.80	0.21

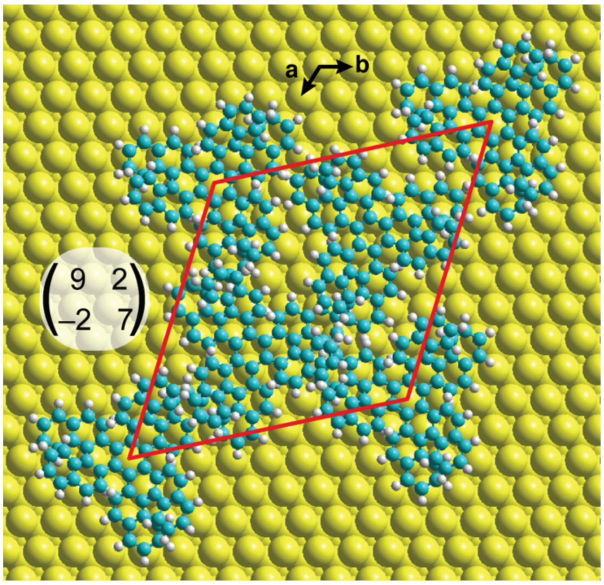

Figure S1. Model of unit cell of one mirror domain. The molecules at the corner are separated by 2.36 nm. The matrix notation is indicated. In Wood notation the unit cell is $(\sqrt{67} \times \sqrt{67})$.

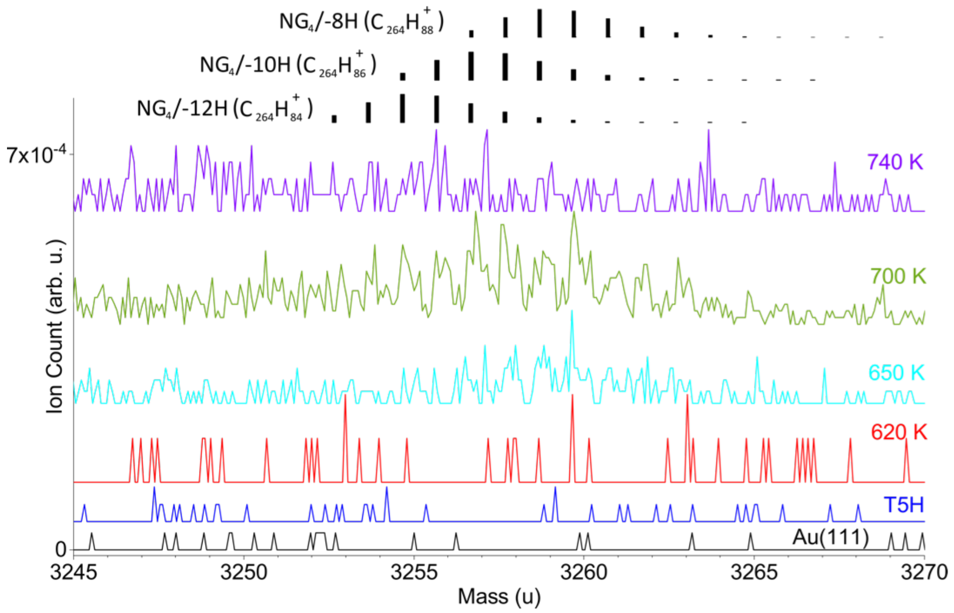

Figure S2. ToF-SIM spectra of the 4 NG region of T5H on Au(111) and after stepwise annealing to the indicated temperatures. Again, several tetramers might contribute to the green spectrum such as NG4/−{8,10,12}H.
